# Female reproduction bears no survival cost in captivity for gray mouse lemurs

**DOI:** 10.1002/ece3.5124

**Published:** 2019-05-18

**Authors:** Julie Landes, Pierre‐Yves Henry, Isabelle Hardy, Martine Perret, Samuel Pavard

**Affiliations:** ^1^ Eco‐Anthropologie, UMR 7206 CNRS, MNHN, Univ. Paris Diderot Paris France; ^2^ Mécanismes Adaptatifs et Evolution (MECADEV ‐ UMR 7179) CNRS, MNHN Brunoy France; ^3^ Département de Biologie, Faculté des Sciences Université de Sherbrooke Sherbrooke Québec Canada

**Keywords:** cost of reproduction, environmental conditions, interindividual heterogeneity, mortality hazard, resources allocation, survival

## Abstract

The survival cost of reproduction has been revealed in many free‐ranging vertebrates. However, recent studies on captive populations failed to detect this cost. Theoretically, this lack of survival/reproduction trade‐off is expected when resources are not limiting, but these studies may have failed to detect the cost, as they may not have fully accounted for potential confounding effects, in particular interindividual heterogeneity. Here, we investigated the effects of current and past reproductive effort on later survival in captive females of a small primate, the gray mouse lemur. Survival analyses showed no cost of reproduction in females; and the pattern was even in the opposite direction: the higher the reproductive effort, the higher the chances of survival until the next reproductive event. These conclusions hold even while accounting for interindividual heterogeneity. In agreement with aforementioned studies on captive vertebrates, these results remind us that reproduction is expected to be traded against body maintenance and the survival prospect only when resources are so limiting that they induce an allocation trade‐off. Thus, the cost of reproduction has a major extrinsic component driven by environmental conditions.

## 1 INTRODUCTION

The cost of reproduction (CoR) is a central mechanism in the evolution of species’ life‐history traits. Because acquired resources are finite, fitness is maximized for an optimal balance between the allocation to current fecundity versus the allocation to survival and future reproduction (Cody, 1966; Reznick, 1985; Stearns, 1989; Williams, 1966). This has proved a robust theory for explaining the difference in life‐history strategies within and between clades (e.g.,Charnov, 2002; Linden & Møller, 1989).

Within species however, the role played by CoR in generating variance in individuals’ life trajectories is yet largely unknown. Experimental procedures—such as artificially increasing reproductive load—generally succeed to evidence costs (see in Boonekamp, Salomons, Bouwhuis, Dijkstra, & Verhulst, 2014; reviewed in Santos & Nakagawa, 2012). But evidencing CoR from longitudinal demographic data has proved difficult. It is particularly the case for evidencing survival CoR (rather than reproductive CoR as in Kroeger, Blumstein, Armitage, Reid, & Martin, 2018) and long‐term CoR (rather than short‐term CoR as in Froy, Walling, Pemberton, Clutton‐Brock, & Loeske, 2016). Even though there is an overall support for trade‐offs between allocating resources to reproduction early in life or to somatic maintenance later in life (Lemaître et al., 2015), some studies failed at detecting these trade‐offs (e.g., 5 out of 26, ibid). When such trade‐offs are detected, it is often not clear if they evidence CoR because many studies consider the link between late‐survival and late recruitment or age at first reproduction rather than its link with actual reproductive effort (e.g., 12 out of 26, ibid). For instance, in black‐legged kittiwakes, a negative relationship was found between breeding (vs. not breeding) and immediate survival (Aubry, Cam, Koons, Monnat, & Pavard, 2011). However, in the same study, a positive relationship between cumulative reproductive effort and late‐survival was evidenced. Consequently, scientists have difficulties to study and estimate these trade‐offs, and wonder whether they exist at all (Metcalf, 2016).

The CoR may be difficult to estimate from longitudinal demographic data for two main reasons. The first reason is that variance in resource acquisition (through the life of an individual or between individuals) may mask variance in resource allocation (Houle, 1991; van Noordwijk & de Jong, 1986). For example, controlling for variance in individual quality allows to take into account the fact that individuals of better quality than the average both reproduce more and live longer. If this heterogeneity is not properly controlled for, type II statistical error may occur, or positive associations between fecundity and survival may even be found (Cam, Link, Cooch, Monnat, & Danchin, 2002; Hamel, Côté, Gaillard, & Festa‐Bianchet, 2009; King, Roff, & Fairbairn, 2011).

The second main reason that makes estimates of CoR difficult is that, as previous studies have suggested, reproduction is costly when, or where, resources are limiting, otherwise reproduction can be completed at no cost in terms of survival, or at a cost that is so weak that we do not have the sufficient statistical power to detect it. In fact, survival CoR has been suggested to depend on environmental conditions: Resources may be limiting only in some years, or at some sites. For example, in Soay sheep, survival CoR was detected only during severe environmental conditions (Tavecchia et al., 2005) or during epizootic outbreaks (Garnier, Gaillard, Gauthier, & Besnard, 2016). Temporary resource limitation can also result from density fluctuations, reproduction being costly only in high‐density years (e.g., Hamel, Côté, & Festa‐Bianchet, 2010). In plants, CoR can be detected or not depending on local climate and length of the growing seasons (Sletvold & Agren, 2015). Last but not least, in captive populations, resources (food, water, mate, and shelter) are generally provided in sufficient quantity so that they do not limit animal welfare, maintenance, and reproduction. Studies on zoo populations for 18 mammal and 12 bird species (Ricklefs & Cadena, 2007), on Rottweiler pet dogs (Kengeri, Maras, Suckow, Chiang, & Waters, 2013) and laboratory mice (Tarin, Gomez‐Piquer, Garcia‐Palomares, Garcia‐Perez, & Cano, 2014), have actually failed to detect any CoR. Positive relationship between reproductive effort and survival was even found in ruffed lemurs kept in zoos (Tidière, Lemaître, Douay, Whipple, & Gaillard, 2017).

Studies on captive populations provide an important complementary set of evidences when analyzing CoR. In captive populations, the major source of noise that compromises the detection of a trade‐off in longitudinal demographic data from wild animals is generally minimized: all sources of heterogeneity in resource acquisition between individuals (either because of spatial structure, temporal fluctuations, or individual heterogeneity) are maintained as low as possible. High standards of animal care generally aim at reducing physiological stress and pathogen exposure, while predation is absent. Comparative studies between captive and free‐ranging populations have therefore proved fundamental in disentangling the extrinsic and intrinsic factors responsible for mortality patterns (Lemaitre, Gaillard, Lackey, Clauss, & Muller, 2013). Housing conditions also often implies providing unrestricted access to food, water, and shelter (ad libitum acquisition) through time, and to all individuals, whatever their characteristics. Captive populations therefore combine reduced noise and life‐history data completeness, which maximizes the chances to detect CoR, if any. However, if CoR is generated by resource limitation, then CoR should not exist in captive animals, at least when animal care aims at alleviating constraints on reproduction and survival (i.e., when the goal is to maintain a sustainable captive population). As discussed above, this could explain the absence of CoR detected in captive species. However, detailed longitudinal data, allowing careful exploration of variance in demographic traits, are necessary to determine whether heterogeneity between individuals also arise in captivity and could compromise the statistical detection of CoR. To take into account interindividual heterogeneity, it is necessary to consider the determinants of these differences between individuals, and thus to incorporate individual variables in the analyses. But because these covariates cannot explain all interindividual variance, it is necessary to also take into account the residual interindividual variability by adding random effects in the analyses (Plard et al., 2015). Former studies did not have detailed individual information (such as health, lineage, or body mass), and thus were not able to unambiguously conclude the absence of CoR in captive populations (Kengeri et al., 2013; Ricklefs & Cadena, 2007; Tarin et al., 2014; Tidière et al., 2017).

Here, we analyzed the potential survival CoR in female captive gray mouse lemurs (*Microcebus murinus*), a small Strepsirrhine primate. All individuals from this population were monitored from birth to death and provided with ad libitum water and shelter availability. Food was delivered in quantities that were sufficiently high to maximize reproduction and survival, while not too abundant to prevent excessive fattening and the negative consequences of long‐term obesity (Terrien et al., 2017). We hypothesize that under such conditions, with nonlimiting resources and virtually no extrinsic mortality, reproducing would not negatively affect future survival. Relative to former similar studies, the strength of our approach was to search for survival CoR while statistically controlling for as many confounding effects as we could, specifically individual heterogeneity, reproductive effort (and its determinants), and biases induced by animal care and management practices.

## 2 MATERIALS AND METHODS

### 2.1 Data

#### 2.1.1 Captivity conditions and reproduction

The analyzed data come from a 16‐year monitoring of a captive population of gray mouse lemurs (see Languille et al., 2012 and Landes et al., 2017 for a detailed description). Animals were kept in monosexual groups of one to five individuals per cage. Average temperature (23–25°C) and humidity (55%) were kept constant. Food and water were nonlimiting and provided in stable quantity all year long. When an individual abnormally lost weight or appeared socially excluded (resting alone or outside from shelter), it was isolated or moved to a new social group, made of individuals of similar body mass. This procedure prevented the establishment of body mass‐driven competitive access to food or shelter. Pathogens (intestinal parasites) were rare and were treated when detected. Wounded and unhealthy individuals were systematically isolated and received veterinary care. Environmental resources can therefore be considered as nonlimiting and extrinsic mortality to be negligible.

Seasonal cycles were induced by changes in day length. The reproductive season was induced by a 6‐month exposure to long days (LD period; 14h of light per day), and the resting season was induced by a 6‐month exposure to short days (SD period; 10h of light per day; Perret & Aujard, 2001). The alternation of LD and *SD* seasons triggers the physiological and behavioral seasonal changes observed in the wild, and mortality fluctuates between high mortality during LD seasons and low mortality during *SD* seasons (Landes et al., 2017).

Reproduction takes place during LD seasons, estrus happening about two weeks after the transition from *SD* to LD season. In the wild, mouse lemurs can reproduce once or twice per reproductive season. In captivity, they were given, if any, only one opportunity to mate per LD season. Reproductive opportunity was under the control of the animal manager (M. Perret) throughout the study period. The main rules for giving an individual the opportunity to breed were as follows: (a) nearly systematic presentation to males for yearling females (respectively 86% at first reproductive seasons, 12% at second, and 2% at later); (b) to favor reproduction of individuals from under‐represented maternal lineages (which refers to the descent from females funders of the population) in an attempt to maintain genetic diversity over the long term; (c) females having failed their first reproduction attempt were usually given the opportunity to breed on the following year, but after 2 or 3 failures, they were definitely removed from females chosen for reproduction; and (d) females showing low weight (a primary indicator or health) or overweighed females were less prone to be chosen to reproduce.

As gray mouse lemurs are polyandrous (Huchard et al., 2012), the males and females selected for reproduction were kept together during the estrus period in groups of six to ten individuals for 2–3 weeks. As a consequence, reproductive success of males is unknown and only females CoR could be investigated in the present study. After this brief mating period, individuals were returned to their monosexual group. After one month, abdominal palpations were performed and females diagnosed to be pregnant were isolated in individual cages until offspring weaning. Gestation is about two months long (60–63 days) and lactation lasts about 40 days (Canale, Huchard, Perret, & Henry, 2012; Perret, 2000).

#### 2.1.2 Data setting and description

The analyzed dataset included 271 captive‐born females, between 1996 and 2011, that had the opportunity to reproduce (successfully or not) at least once over their lifetime. All females entered the study at their first breeding opportunity. Mortality of captive female gray mouse lemurs fluctuates in a large extent between seasons, with 75% of deaths occurring in LD seasons (Landes et al., 2017). Because both reproduction and mortality largely occurred during the LD seasons, we restricted our analyses to LD seasons. To do this, survival data were left‐truncated at the entrance of the individuals in each new LD season and, if the individuals did not die or exited the study (censoring) within the season, the data were right‐censored at the end of the season (as in Landes et al., 2017). Because females that successfully reproduced during a given LD season were alive at the delivery, their survival data were left‐truncated at this date and mortality was strictly analyzed afterward until the end of the season.

In total, our sample incorporated 812 season individuals of adult females surviving until their first breeding season. Of these, 77 individuals experienced natural death during a LD season (animals found dead or about to die) that occurred between 8 months and 7.63 years of age (mean 3.76 ± 1.65). The other 194 individuals were censored (30 died because of accidents or experimental procedures or were transferred to other captive facilities, 26 died of natural death during a *SD* season, 10 were censored during a *SD* season, and 128 were still alive at the end of the study, that is, January 1st, 2013). All females had the opportunity to breed (successfully or not) at least once during the study period for a total of 399 recorded breeding opportunities leading to 258 reproductive successes and 141 breeding failures (unsuccessful fecundation or early abortion). The females had one to five opportunities to breed over their lifetime (1.47 ± 0.75), mostly at young ages (90% of breeding opportunities occurred before the age of 3.5 years).

About 70% of the variance in breeding opportunity (i.e., the probability to have an opportunity to reproduce) could be explained by measured variables characterizing the rules set by the manager of the captive population: the number of breeding opportunities provided by the population manager by year, females’ age, body mass, past reproductive failures, and maternal lineage (see Appendix S1 for logistic regressions). Interestingly, no effect of past reproductive failure on further breeding opportunity has been detected for females having successfully reproduced at least once: for females that had at least one reproductive success, breeding opportunity was independent of previous reproductive success.

By contrast, only 28% of the variance in reproductive success was explained by measurable variables (mainly age, maternal lineage, and failing first reproduction) and the factors responsible for reproductive success (vs. failure) remained largely unknown. Period or cohort effects, fluctuation in mass, age at first reproduction, or past reproductive success failed to statistically explain variance in reproductive success.

### 2.2 Survival analyses

To determine if reproduction compromises female mortality, we used semiparametric proportional models (also called Cox's model; Cox, 1972; Klein & Moeschberger, 2003) using the “coxph” function in the “survival” library of “R” (R‐Development‐Core‐Team, 2011; Therneau & Grambsch, 2000). Cox models are flexible and do not constrain the distribution of the baseline hazard, but they assume that the effect of a covariate is proportional to the baseline mortality level at all ages (see Appendix S2).

We used two sets of models as analytical strategy. A first set of models (Preliminary Runs) was designed to analyze the effects of past and current reproductive success (respectively *CumRS* and *RS* in Table 1) on female mortality and to identify the adjustment variables affecting female mortality: (a) current and past breeding opportunities (variables 13–18 in Table 1) and the interaction between past breeding opportunities and reproductive success (*CumBO:CumRS*), (b) body mass (an indicator of individual's health) and its variation over time (variables 19–21 in Table 1), (c) maternal lineage (variables 22–23 in Table 1), and (d) cohort and period effects (variables 24–25 in Table 1).

**Table 1:** Description of the variables of interest. Unless specified, summary statistics are mean ± *SD* Minimal–maximal values are reported in the main text

	Name	Notation		Description	Type	Statistic
Variable describing current reproductive success						
1	Reproductive success	*RS*	Successful reproduction in a LD season (0 = No; 1 = At least one offspring delivered). Defined only for *BO* = 1		Boolean. Time‐Varying	0.64 ± 0.47
Variables describing current reproductive effort for successful reproductions^a^						
2	Litter size	*LitterSize*	# of offspring born in a given reproductive event. Defined only for *RS* = 1.		Continuous, Time‐Varying	2.1 ± 0.73
3	Sex ratio	*SexRatio*	Ratio of the number of males to the number of offspring. Defined only for *RS* = 1.		Continuous, Time‐Varying	0.51 ± 0.36
4	# of males	*NbMales*	# of males in the litter. Defined only for *RS* = 1.		Continuous, Time‐Varying	1.06 ± 0.77
5	# of deaths	*Deaths*	# of offspring dying before weaning in the litter. Defined only for *RS* = 1.		Continuous, Time‐Varying	0.22 ± 0.55
6	Neonatal mortality	*Mortality*	Litter mortality rates between birth and weaning. Defined only for *RS = *1.		Continuous, Time‐Varying	0.11 ± 0.27
Variable describing past reproductive successes						
7	Cumulative reproductive success	*CumRS*	# of past reproductive successes.		Continuous, Time‐Varying	0.95 ± 0.71 b
Variables describing past reproductive effort a						
8	Cumulative litter size	*CumLitterSize*	Cumulative # of offspring over past reproductions.		Continuous, Time‐Varying	2.00 ± 1.81 b
9	Mean sex ratio	*MeanSexRatio*	Mean sex ratio over past reproductions (set to 0.5 if no past reproductive success).		Continuous, Time‐Varying	0.51 ± 0.30 b
10	Cumulative # of males	*CumNbMales*	Cumulative # of males produced over past reproductions.		Continuous, Time‐Varying	1.01 ± 1.11 b
11	Cumulative # of deaths	*CumDeaths*	Cumulative # of offspring dying before weaning over past reproductions.		Continuous, Time‐Varying	0.21 ± 0.58 b
12	Mean neonatal mortality	*MeanMortality*	Mean neonatal mortality over past reproductions (set to 0.1, i.e., mean mortality, if no past reproductive success).		Continuous, Time‐Varying	0.10 ± 0.20 b
Variables describing current and past breeding opportunities						
13	Breeding opportunity	*BO*	Opportunity to reproduce in a LD season (0 = No; 1 = Yes).		Boolean. Time‐Varying	0.49 ± 0.50
14	# of past breeding opportunities	*CumBO*	Cumulative # of past breeding opportunities.		Continuous, Time‐Varying	1.47 ± 0.74 b
15	Failure of first reproduction	*Failure1^st^*	Failure of first reproduction (0 = success; 1 = failure).		Boolean. Fixed	0.25 ± 0.43
16	No past reproductive success	*NoSuccess*	No past reproductive success (0 = past success; 1 = no past success).		Boolean. Time‐Varying	0.20 ± 0.40
17	First breeding opportunity	*FirstBO*	LD season of first breeding opportunity at which females entered the study; respectively first, second, or later.		Factorial, Fixed	*n* = 235, 29 and 7
18	Opportunity to breed by year	*BOYear*	Chances of having the opportunity to breed a given year, calculated as the # of breeding opportunities divided by the # of living females at the beginning of the LD season of a given year. Account for a pseudo‐“Density” effect because population size is maintained constant and, although there is no competition for resources, the # of breeding opportunities per year is limited to maintain the population within housing capacity.		Continuous, Time‐Varying	0.43 ± 0.26
Adjusting variables						
19	Body mass	*Mass*	Mass at the entrance into the season^c^ (in grams).		Continuous, Time‐Varying	85 ± 16
20	Body mass variation between reproductive seasons	*MassVar*	Relative mass compared to the one at last breeding opportunity (set to 1 for the first *BO*)^c^. It documents potential gain or loss of mass compared to the last time the female has been judged fit for reproduction.		Continuous, Time‐Varying	1.1 ± 0.21
21	Short‐term loss in body mass	*MassLoss*	Relative mass compared to the last *SD* or LD season^c^. It documents sudden loss in mass (>1 if mass declines).		Continuous, Time‐Varying	0.96 ± 0.17
22	Maternal lineage	*Lineage*	It indicates from which of the six initial founder females a given female is descending from. It documents potential variations arising from (epi)genetic inheritance through females		Factorial, Fixed	*n* = 26, 62, 25, 45, 13, 100
23	Frequency of maternal lineages per year	*FreqLineage*	Frequency of a given maternal lineage per year calculated at the level of the whole colony including females not incorporated into the present analysis (*n* = 1765 females living between 1997 and 2012). It documents the tendency to favor breeding of lowest frequency lineages.		Continuous, Time‐Varying	0.24 ± 0.14
24	Cohort effect on mortality	*YearBirth*	Year at which a given female is born (1996–2011). It documents potential cohort effects on mortality. For survival analysis, the variable is clustered^d^ in three groups ranging from low to high effect on mortality.		Factorial, Fixed	
25	Period effect on mortality	*YearObs*	Year at which the given season occurs (1997–2012). It documents potential period effects on mortality. For survival analysis, the variable is clustered^d^ in three groups ranging from low to high effect on mortality.		Factorial, Time‐Varying	
26	Interindividual heterogeneity	*Identity*	Individual identity—Random intercept for each female.		Random variable	
27	Maternal effect	*Mother*	Mother identity—Random intercept for all sibling females.		Random variable	

a Because the number of males in a litter was the product between litter size and its sex ratio (LitterSize*SexRatio = *NbMales*), redundancy forbade to incorporate the three variables into the same model. Rather, we tested for an additive effect of the number of offspring born (*LitterSize*) together with either an absolute (*NbMales*) or relative (*SexRatio*) effect of the number of males in the litter. A similar reasoning was applied for (a) *CumLitterSize*, *CumNbMales* and *MeanSexRatio*; (b) *LitterSize*, *Deaths* and *Mortality*; and (c) *CumLitterSize*, *CumDeaths* and *MeanMortality*.

b Calculated at last season lived for time‐varying variables, whether individuals’ follow‐up is interrupted by death or censoring.

c Body mass was unknown for 9 out of the 1802 female.seasons of the dataset and were estimated by the mean individual mass at the entrance in LD seasons.

d Accounting for year of birth or observation proved important to adjust for uncontrolled temporal variations in survival in the studied population (Landes et al., 2017). However, a model assuming that all years and all cohorts are fully independent would lead to convergence issues and a loss of a statistical power. To overcome this, we grouped years according to their relative risks of death (as previously done in Aubry et al., 2011 and Landes et al., 2017). The effect of year at observation (*df *= 15) and year of birth (*df *= 14) on individual survival was estimated using Cox survival analyses. We then used the models’ estimates to cluster the years (using “pam” function of the “cluster” package in “R”) into 3 groups explaining changes in survival in the most parsimonious fashion.

A second set of models (CoR Runs) was designed to investigate which components of past and current reproductive successes and efforts (litter size, litter’ sex ratio, and litter's mortality), may have an effect on female mortality, if any (variables 2–6 and 8–12 in Table 1). Models of CoR Runs incorporated the adjustment variables selected during Preliminary Runs (see Appendix S3).

For each model, the proportionality assumption (*p* > 0.05) was checked using the “cox.zph” procedure (“survival” library of “R”). Model selection was based on Akaike's Information Criterion corrected for small sample size (AICc; with number of observations being 77 natural deaths; Akaike, 1974; Burnham & Anderson, 1998); and the highest ranked models were considered (ΔAICc < 2). We investigated potential hidden individual heterogeneity (frailty or maternal effect) by incorporating the identity of the individual and its mother as random effects (variables 26–27 of Table 1). Because AIC values are not compelling for comparing models including random variables (Jiang, Rao, Gu, & Nguyen, 2008), we compared the magnitude and significance of the coefficients estimated for the fixed variables using models including, or not, these random variables. To the best of our knowledge, there is no available method to estimate type II statistical error for survival analyses. Hence, we designed an ad hoc method to estimate how many deaths would have been necessary to detect CoR, if present, in the present dataset (Appendix S2).

## 3 RESULTS

All results presented hereafter are consistent throughout statistical analyses, with a low probability of type II statistical error (see Appendix S2).

CoR Runs included all the 5,183 models that incorporated *FirstBO, FreqLineage, YearBirth, *and *Failure1th* and all the combinations between *CumRS, RS*, *Rmass*, *BOperYear, *and the 10 variables describing the characteristics of current and past reproductions (see variable definitions in Table 1; results from Preliminary Runs and selection of variables for CoR Runs are detailed in Appendix S3). All models converged and validated the mortality hazard proportionality assumption.

No cost of reproduction was evidenced by our analyses, which controlled for potential confounding effects. Table 2 presents the highest AICc‐ranked models together with the least and most parameterized models among the 44 ones with ΔAICc* < *2. Level of offspring mortality and failure of first reproduction were significantly linked to larger mortality. Producing offspring, however, did not compromise female survival. Our results were the same independently to the number of parameters and the random effects included in the models. Indeed, effects were robust in magnitude and significance to the number of parameters included in the models. Adding random variables accounting for frailty or maternal effect to the highest ranked model did not change the magnitude or significance of the other variables and their effects were found nonsignificant (p‐values, respectively, 0.93 and 0.93). Schoenfeld residuals plotted over age for each of the covariates showed no deviation from the proportional hypothesis.

**Table 2:** Effect of past and current reproductive success and effort on female mortality

*RS*	LitterSize^a^	Deaths^a^	CumLitterSize	CumDeaths	MassVar	BOYear	FirstBO	FreqLineage	Failure1th	YearBirth	AICc	ΔAICc
Highest ranked model												
‐	−1.67^*^	2.28^*^	−0.15 ns	0.495^*^	‐	‐	−0.37 ns −2.05^†^	−1.97^*^	0.57^†^	−0.09 ns ‐16.01 ns	613.70	0
Least parameterized model among the highest ranked model												
‐	−1.57^†^	2.29 ^*^	‐	‐	‐	‐	−0.29 ns −1.95^†^	−1.69 ^*^	0.80 ^*^	0.10 ns −16.31 ns	613.88	0.17
Most parameterized model among the highest ranked model												
1.336 ns	−2.53^*^	2.48^*^	−0.169^†^	0.533^*^	−1.07^†^	−0.83^†^	−0.42 ns −1.83^†^	−1.84^*^	0.56 ns	0.31 ns −15.78 ns	615.56	1.86
Averaged effects												
1.53 ns	−1.84^*^	2.31^*^	−0.14^†^	0.47^*^	−0.90 ns	−0.75^†^	−0.32 ns 1.90 ^†^	−1.85^*^	0.72^†^	0.14 ns −16.09 ns		
Highest ranked model for individuals that have succeed at least one reproduction (CumLitterSize > 0, *n* = 173 individuals over 439 seasons)												
	−1.19 ns	1.95^*^	−0.18^†^	0.47^†^			−0.58 ns 1.73 ns	−1.61^†^	0.87^*^	0.03 ns −15.82 ns		

Notes

Variables are defined in Table 1.

Statistical significance is indicated by ns for *p* > 0.1; “^†^” for *p* < 0.1; “^*^” for *p* < 0.05.

a Entered in interaction with *RS *(e.g., *RS:LitterSize*).

Contrary to the CoR hypothesis, our results showed that producing large litters tended to decrease females’ mortality during the considered reproductive season. However, martingale residuals plotted against the continuous variables (not shown) demonstrate problems in the functional distribution of the *LitterSize* variable. This is due to the higher mortality of females that had never succeeded at least one successful reproduction and selecting only individuals that successfully reproduce at least once make the protecting effect of *LitterSize* disappear (see Table 2).

**Figure 1:**
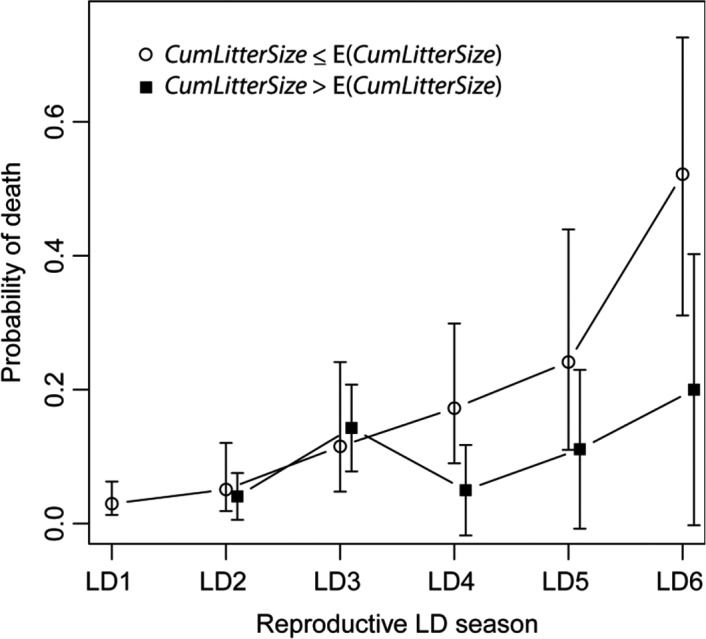
Probability of death over a reproductive season according to whether females have produced larger (black square) or a lower or equal (white circle) number of offspring than the average [E(*CumLitterSize*)] for their counterparts of same age. “LD” stands for “Long Days.” High reproductive effort tends to decrease mortality, particularly after 3‐year old

At the opposite to CoR theory, reproduction could even be beneficial to survival. All highest ranked models evidenced a trend for past cumulated litter size (*CumLitterSize*) to decrease female mortality. This trend—a decrease in about 15% of further mortality by offspring produced (see also Figure 1)—remained particularly robust trough models, even when focusing only on the individuals that have successfully reproduced at least once in their lifetime. It must be stressed that these results (i.e., reproduction is not associated with increased mortality early in life but rather tend to increase survival at older ages) are also visible from raw estimates (see Figure 1).

## 4 DISCUSSION

In our captive population of gray mouse lemurs, we could not evidence any survival cost of reproduction (CoR) for females. Rather, a trend toward lower mortality of females with increased cumulated reproductive effort was evidenced both during and after the reproductive event (Table 2, Figure 1). These results have been obtained from longitudinal, continuous birth‐to‐death monitoring data from individuals maintained under controlled and constant environmental conditions, with nonlimiting resource availability. Furthermore, this dataset allowed us to control for the well‐known confounding effect of heterogeneity in breeding success across individuals that tends to hide CoR. (see below and Appendix S1). Our statistical approach also minimized the risk of not detecting an existing CoR as we used appropriate proxies of reproductive effort, and we controlled for several potential confounding effects. We also determined—using an ad hoc method (Appendix S2)—that type II statistical error was not likely affecting our results.

Former studies on other captive populations of vertebrates reached the same conclusion: no evidence of survival cost of reproduction could be found (Kengeri et al., 2013; Ricklefs & Cadena, 2007; Tarin et al., 2014; Tidière et al., 2017). For instance, Tidière et al., 2017 failed to detect a negative impact of reproduction on survival using a sample of 5,358 ruffed lemurs kept in a zoo. However, all referred former studies (including Tidière et al., 2017) had little opportunity for properly controlling for variance in resource acquisition, either through time or between individuals. The present study does control for various confounding variables and suggests that the lack of CoR in former studies is actually a true lack of CoR and not a spurious conclusion due to hidden sources of heterogeneity that had not been properly accounted for in statistical analyses. Although we used a lower number of individuals than the aforementioned previous studies, their life histories were much more detailed, and allowed the examination of many alternative facets (i.e., variables) of the potential cost of reproduction, while accounting for many possible sources of variance in resources acquisition between individuals (using individual covariates for deterministic effects, and random variables for unexplained residual between‐individual variation). Noticeably, we controlled for: (a) a well‐known cofounding factor when estimating CoR in the wild: the fact that heterogeneity in individuals’ quality may translate into variance in breeding opportunity. In this case, robust individuals are more likely to reproduce and survive than frail ones. Here, breeding opportunity was determined by the population manager, and 70% of its variance could be explained by measurable variables (see Appendix S1), which were included in the analyses. (b) Past reproductive success, as we demonstrated that failing at the first reproduction opportunity compromised both further reproduction and survival. (c) Fluctuation of body mass throughout the females’ life, as sudden loss in mass or overweight compromises survival. (d) Period and cohort effects. (e) Unobserved interindividual heterogeneity and maternal effect.

Our results suggest that Darwinian demons (Law, 1979) may not be only phantasmagorical creatures, at least on the short term: Wild animals maintained under unlimited resources in a predator‐ and parasite‐free environment can become such Darwinian demons. The results of our analysis are coherent with previous studies that fail to evidence CoR in captive populations of birds and mammals (Ricklefs & Cadena, 2007; Tarin et al., 2014). When resources are abundant and predation and parasitism are absent, the individual seems to be able to reproduce without the need to compromise allocation to organism maintenance and survival. These results show that trade‐offs are condition‐dependent, shedding new light on the hidden structure of acquisition‐allocation trade‐offs. This is first because variance in resource acquisition (van Noordwijk & de Jong, 1986) must therefore encompass the heterogeneity in individual quality but also the fluctuation of resources available through time and their interactions (as suggested by Ricklefs & Cadena, 2007). This is second because the slope of the trade‐off between reproduction and survival is also likely condition‐dependent, meaning that, over time, individuals fluctuate between state of large and low survival and fertility. Further study should therefore consider survival CoR as a phenotype subject to a reaction norm in response to environmental conditions (as suggested by Hamel et al., 2010). This may also explain the difficulty to disentangle the genetic and physiological components of the covariance between demographic traits (Agrawal, Conner, & Rasmann, 2010; Conner, 2012).

In captive gray mouse lemurs, reproduction seems to even increase survival. Individuals reproducing more than twice died half less than individuals successfully reproducing once. This result held until old ages. This positive association between reproductive success and survival may have some physiological ground. Indeed, female reproductive hormones are known to have a protective effect on several biological functions, and therefore potentially, on organismal maintenance. For instance, estrogen exposure—and its endocrine complement progesterone—decreases neurodegeneration and would protect against stroke (Garcia‐SeguraAzcoitia & DonCarlos, 2001; Singh, 2006). Reproduction would decrease the risk of breast cancer and increases the chances of surviving a breast cancer at old ages (Thalib, Doi, & Hall, 2005). It would also effectively protect the organism against the damages of the burst of oxidative stress induced by the reproductive effort, potentially through an improved efficiency of the antioxidant response (Ołdakowski et al., 2012). The oxidative status of reproductive females can even be better than the one of nonreproductive females (Garratt et al., 2011). Overall, these studies suggest that, in female mammals, reproduction could have a protective effect on several physiological functions closely linked with the survival prospects.

To conclude, our study evidenced no survival cost of reproduction in a captive population of gray mouse lemurs. Our conclusions are the most robust possible given the available sample size as we controlled for confounding variables (determinants of breeding opportunity, proxies of individual quality, cohort, and period effects), used multiple reproductive investment variables, and secured a sufficient statistical power. Such detailed data and carefully controlled analysis are used for the first time in the context of survival CoR estimation, and we recommend using it to secure inference robustness in future studies. Overall, our results emphasize the fact that the CoRs are environment‐dependent. Resource availability does influence the allocation strategy and must be limiting for reproduction to be successful at the expense of the survival prospect. Otherwise, no survival CoR is expected.

## CONFLICT OF INTEREST

The authors have no conflicts of interest to declare.

## AUTHORS’ CONTRIBUTIONS

J.L., S.P., and P.‐Y.H. jointly conceived the original study. M.P. supervised the data collection process and captive population management. I.H. created and updated the database. S.P. conducted the analyses together with J.L. J.L., S.P., and P.‐Y.H. interpreted the results and wrote the manuscript.

## Data Availability

The data supporting the results are archived on Dryad (https://doi.org/10.5061/dryad.g860c9s).

## References

[ece35124-bib-0001] Agrawal, A. A. , Conner, J. K. , & Rasmann, S. (2010). Tradeoffs and negative correlations in evolutionary ecology In Bell M. A., Eanes W. F., Futuyma D. J., & Levinton J. S. (Eds.), Evolution after Darwin: The first 150 years. Sunderland, MA: Sinauer Associates.

[ece35124-bib-0002] Akaike, H. (1974). A new look at the statistical model identification. IEEE Transactions on Automatic Control, 19(6), 716–723. 10.1109/tac.1974.1100705

[ece35124-bib-0003] Aubry, L. M. , Cam, E. , Koons, D. N. , Monnat, J. Y. , & Pavard, S. (2011). Drivers of age‐specific survival in a long‐lived seabird: Contributions of observed and hidden sources of heterogeneity. Journal of Animal Ecology, 80(2), 375–383. 10.1111/j.1365-2656.2010.01784.x 21182519

[ece35124-bib-0004] Boonekamp, J. J. , Salomons, M. , Bouwhuis, S. , Dijkstra, C. , & Verhulst, S. (2014). Reproductive effort accelerates actuarial senescence in wild birds: An experimental study. Ecology Letters, 17(5), 599–605. 10.1111/ele.12263 24818237

[ece35124-bib-0005] Burnham, K. P. , & Anderson, D. R. (1998). Model selection and inference: A practical information‐theoretic approach. New York, MA: Springer‐Verlag.

[ece35124-bib-0006] Cam, E. , Link, W. A. , Cooch, E. G. , Monnat, J.‐Y. , & Danchin, E. (2002). Individual covariation in life‐history traits: Seeing the trees despite the forest. The American Naturalist, 159(1), 96–105. 10.1086/324126 18707403

[ece35124-bib-0007] Canale, C. I. , Huchard, E. , Perret, M. , & Henry, P.‐Y. (2012). Reproductive resilience to food shortage in a small heterothermic primate. PLoS One, 7(7), e41477 10.1371/journal.pone.0041477 22848507 PMC3405090

[ece35124-bib-0008] Charnov, E. L. (2002). Reproductive effort, offspring size and benefit‐cost ratios in the classification of life histories. Evolutionary Ecology Research, 4(5), 749–758.

[ece35124-bib-0009] Cody, M. L. (1966). A general theory of clutch size. Evolution, 20(2), 174–184. 10.2307/2406571 28563630

[ece35124-bib-0010] Conner, J. K. (2012). Quantitative genetic approaches to evolutionary constraint: How useful? Evolution, 66(11), 3313–3320. 10.1111/j.1558-5646.2012.01794.x 23106699

[ece35124-bib-0011] Cox, D. R. (1972). Regression models and life‐tables. Journal of the Royal Statistical Society Series B (Methodological), 34(2), 187–220. 10.1007/978-1-4612-4380-9_37

[ece35124-bib-0012] Froy, H. , Walling, C. A. , Pemberton, J. M. , Clutton‐Brock, T. H. , & Loeske, L. E. B. (2016). Relative costs of offspring sex and offspring survival in a polygynous mammal. Biology Letters, 12(9), 20160417 10.1098/rsbl.2016.0417 27601725 PMC5046923

[ece35124-bib-0013] Garcia‐Segura, L. M. , Azcoitia, I. , & DonCarlos, L. L. (2001). Neuroprotection by estradiol. Progress in Neurobiology, 63, 29–60. 10.1016/s0301-0082(00)00025-3 11040417

[ece35124-bib-0014] Garnier, A. , Gaillard, J. M. , Gauthier, D. , & Besnard, A. (2016). What shapes fitness costs of reproduction in long‐lived iteroparous species? A case study on the Alpine ibex. Ecology, 97(1), 205–214. 10.1890/15-0014.1 27008789

[ece35124-bib-0015] Garratt, M. , Vasilaki, A. , Stockley, P. , McArdle, F. , Jackson, M. , & Hurst, J. L. (2011). Is oxidative stress a physiological cost of reproduction? An experimental test in house mice. Proceedings of the Royal Society B, 278, 1098–1106. 10.1098/rspb.2010.1818 20926440 PMC3049035

[ece35124-bib-0016] Hamel, S. , Côté, S. D. , & Festa‐Bianchet, M. (2010). Maternal characteristics and environment affect the costs of reproduction in female mountain goats. Ecology, 91(7), 2034–2043. 10.1890/09-1311.1 20715626

[ece35124-bib-0017] Hamel, S. , Côté, S. D. , Gaillard, J.‐M. , & Festa‐Bianchet, M. (2009). Individual variation in reproductive costs of reproduction: High‐quality females always do better. Journal of Animal Ecology, 78(1), 143–151. 10.1111/j.1365-2656.2008.01459.x 18700872

[ece35124-bib-0019] Houle, D. (1991). Genetic covariance of fitness correlates: What genetic correlations are made of and why it matters. Evolution, 45(3), 630–648. 10.2307/2409916 28568816

[ece35124-bib-0020] Huchard, E. , Canale, C. I. , Le Gros, C. , Perret, M. , Henry, P.‐Y. , & Kappeler, P. M. (2012). Convenience polyandry or convenience polygyny? Costly sex under female control in a promiscuous primate. Proceedings of the Royal Society of London Series B‐Biological Sciences, 279:1371–1379. 10.1098/rspb.2011.1326 PMC3282357 21976684

[ece35124-bib-0021] Jiang, J. , Rao, J. S. , Gu, Z. , & Nguyen, T. (2008). Fence methods for mixed model selection. The Annals of Statistics, 36(4), 1669–1692. 10.1214/07-aos517

[ece35124-bib-0022] Kengeri, S. S. , Maras, A. H. , Suckow, C. L. , Chiang, E. C. , & Waters, D. J. (2013). Exceptional longevity in female Rottweiler dogs is not encumbered by investment in reproduction. Age (Dordr), 35(6), 2503–2513. 10.1007/s11357-013-9529-8 23584889 PMC3825016

[ece35124-bib-0023] King, E. G. , Roff, D. A. , & Fairbairn, D. J. (2011). Trade‐off acquisition and allocation in *Gryllus firmus*: A test of the Y model. Journal of Evolutionary Biology, 24(2), 256–264. 21044204 10.1111/j.1420-9101.2010.02160.x

[ece35124-bib-0024] Klein, J. P. , & Moeschberger, M. (2003). Survival analysis: Techniques for censored and truncated data. New York: Springer.

[ece35124-bib-0025] Kroeger, S. B. , Blumstein, D. T. , Armitage, K. B. , Reid, J. M. , & Martin, J. G. A. (2018). Cumulative reproductive costs on current reproduction in a wild polytocous mammal. Ecology and Evolution, 8(23), 11543–11553. 10.1002/ece3.4597 30598755 PMC6303762

[ece35124-bib-0026] Landes, J. , Perret, M. , Hardy, I. , Camarda, C.‐G. , Henry, P.‐Y. , & Pavard, S. (2017). State transitions: A major mortality risk for seasonal species. Ecology Letters, 20(7), 883–891. 10.1111/ele.12785 28635125

[ece35124-bib-0027] Languille, S. , Blanc, S. , Blin, O. , Canale, C. I. , Dal‐Pan, A. , Devau, G. , … Aujard, F. (2012). The grey mouse lemur: A non‐human primate model for ageing studies. Ageing Research Reviews, 11(1), 150–162. 10.1016/j.arr.2011.07.001 21802530

[ece35124-bib-0028] Law, R. (1979). Optimal life histories under age‐specific predation. The American Naturalist, 114(3), 399–417. 10.1086/283488

[ece35124-bib-0029] Lemaître, J.‐F. , Berger, V. , Bonenfant, C. , Douhard, M. , Gamelon, M. , Plard, F. , & Gaillard, J.‐M. (2015). Early‐late life trade‐offs and the evolution of ageing in the wild. Proceedings of the Royal Society B: Biological Sciences, 282(1806), 20150209 10.1098/rspb.2015.0209 PMC4426628 25833848

[ece35124-bib-0030] Lemaitre, J. F. , Gaillard, J.‐M. , Lackey, L. B. , Clauss, M. , & Muller, D. W. (2013). Comparing free‐ranging and captive populations reveals intra‐specific variation in aging rates in large herbivores. Experimental Gerontology, 48(2), 162–167. 10.1016/j.exger.2012.12.004 23261518

[ece35124-bib-0031] Linden, M. , & Møller, A. P. (1989). Cost of reproduction and covariation of life history traits in birds. Trends in Ecology & Evolution, 4(12), 367–371. 10.1016/0169-5347(89)90101-8 21227380

[ece35124-bib-0032] Metcalf, C. J. (2016). Invisible trade‐offs: Van Noordwijk and de Jong and life‐history evolution. The American Naturalist, 187(4), iii–v. 10.1086/685487 27028085

[ece35124-bib-0033] Ołdakowski, L. , Piotrowska, Z. , Chrząścik, K. M. , Sadowska, E. T. , Koteja, P. , & Taylor, J. R. E. (2012). Is reproduction costly? No increase of oxidative damage in breeding bank voles. The Journal of Experimental Biology, 215, 1799–1805. 10.1242/jeb.068452 22573758

[ece35124-bib-0034] Perret, M. (2000). Reproduction saisonnière et succès reproducteur chez un prosimien malgache, *Microcebus murinus* . Primatologie, 3, 45–84.

[ece35124-bib-0035] Perret, M. , & Aujard, F. (2001). Regulation by photoperiod of seasonal changes in body mass and reproductive function in gray mouse lemurs (*Microcebus murinus*): Differential responses by sex. International Journal of Primatology, 22, 5–24.

[ece35124-bib-0036] Plard, F. , Gaillard, J.‐M. , Coulson, T. , Delorme, D. , Warnant, C. , Michallet, J. , … Bonenfant, C. (2015). Quantifying the influence of measured and unmeasured individual differences on demography. Journal of Animal Ecology, 84, 1434–1445. 10.1111/1365-2656.12393 26140296 PMC5642278

[ece35124-bib-0037] R‐Development‐Core‐Team. (2011). R: A language and environment for statistical computing. Vienna, Austria: R Foundation for Statistical Computing.

[ece35124-bib-0038] Reznick, D. (1985). Costs of reproduction: An evaluation of the empirical evidence. Oikos, 44(2), 257–267. 10.2307/3544698

[ece35124-bib-0039] Ricklefs, R. E. , & Cadena, C. D. (2007). Lifespan is unrelated to investment in reproduction in populations of mammals and birds in captivity. Ecology Letters, 10(10), 867–872. 10.1111/j.1461-0248.2007.01085.x 17845285

[ece35124-bib-0040] Santos, E. S. A. , & Nakagawa, S. (2012). The costs of parental care: A meta‐analysis of the trade‐off between parental effort and survival in birds. Journal of Evolutionary Biology, 25(9), 1911–1917. 10.1111/j.1420-9101.2012.02569.x 22830387

[ece35124-bib-0041] Singh, M. (2006). Progesterone‐induced neuroprotection. Endocrine, 29(2), 271–274. 10.1385/endo:29:2:271 16785602

[ece35124-bib-0042] Sletvold, N. , & Agren, J. (2015). Climate‐dependent costs of reproduction: Survival and fecundity costs decline with length of the growing season and summer temperature. Ecology Letters, 18(4), 357–364. 10.1111/ele.12417 25711515

[ece35124-bib-0043] Stearns, S. C. (1989). Trade‐offs in life‐history evolution. Functional Ecology, 3, 259–268. 10.2307/2389364

[ece35124-bib-0044] Tarin, J. J. , Gomez‐Piquer, V. , Garcia‐Palomares, S. , Garcia‐Perez, M. A. , & Cano, A. (2014). Absence of long‐term effects of reproduction on longevity in the mouse model. Reproductive Biology and Endocrinology, 12(1), 84 10.1186/1477-7827-12-84 25159296 PMC4156647

[ece35124-bib-0045] Tavecchia, G. , Coulson, T. , Morgan, B. J. T. , Pemberton, J. M. , Pilkington, J. C. , Gulland, F. M. D. , & Clutton‐Brock, T. H. (2005). Predictors of reproductive cost in female Soay sheep. Journal of Animal Ecology, 74(2), 201–213. 10.1111/j.1365-2656.2005.00916.x

[ece35124-bib-0046] Terrien, J. , Gaudubois, M. , Champeval, D. , Zaninotto, V. , Roger, L. , Riou, J. F. , & Aujard, F. (2017). Metabolic and genomic adaptations to winter fattening in a primate species, the grey mouse lemur (*Microcebus murinus*). International Journal of Obesity (London), 42(2), 221–230. 10.1038/ijo.2017.195 28925409

[ece35124-bib-0047] Thalib, L. , Doi, S. A. , & Hall, P. (2005). Multiple births and breast cancer prognosis: A population based study. European Journal of Epidemiology, 20(7), 613–617. 10.1007/s10654-005-5530-6 16119435

[ece35124-bib-0048] Therneau, T. M. , & Grambsch, P. M. (2000). Modeling survival data: Extending the Cox model. New York: Springer.

[ece35124-bib-0049] Tidière, M. , Lemaître, J.‐F. , Douay, G. , Whipple, M. , & Gaillard, J.‐M. (2017). High reproductive effort is associated with decreasing mortality late in life in captive ruffed lemurs. American Journal of Primatology, 79(9), e22677 10.1002/ajp.22677 28608982

[ece35124-bib-0050] van Noordwijk, A. J. , & de Jong, G. (1986). Acquisition and allocation of resources: Their influence on variation in life history tactics. The American Naturalist, 128(1), 137–142. 10.1086/284547

[ece35124-bib-0051] Williams, G. C. (1966). Natural selection, the costs of reproduction, and a refinement of Lack's principle. The American Naturalist, 100, 687–690. 10.1086/282461

