# Supplementary material for: Female reproduction bears no survival cost in captivity for gray mouse lemurs
Source: Ecol Evol. 2019 May 18;9(11):6189–98. doi: 10.1002/ece3.5124 (PMC6580269; doi:10.1002/ece3.5124)
Supplement: Supplementary file 1 [file ECE3-9-6189-s001.docx]

**Appendix 1 - Determinants of breeding opportunity and success**

**Methods**

We used generalized linear (mixed) logistic regressions to analyse the determinants of breeding opportunity (i.e. the probability that a female will be given the opportunity to breed) and reproductive success (i.e. the probability that a female having the opportunity to breed will produce offspring; see variables *BO* and *RS* in Table 1).

Because breeding opportunity occurs in LD seasons only, analysis of *BO* was performed with the 812 females-LD-seasons of the dataset. Analysis of *RS* was performed with 399 females-LD-seasons having the opportunity to breed a given year. More precisely, analyses explored first the determinants of *RS* at first opportunity to breed (n=271) and then for subsequent opportunity to breed (n=128). The covariates incorporated into the analyses were females’ age (denoted *Age*), variables describing past breeding opportunities and reproductive successes (variables 7 and 14 in Table 1) and fluctuations in mass (variables 19-21 in Table 1). Adjustment variables were also incorporated in the models to account, for example, for period, cohort, or ‘pseudo-density’ effects (variables 15-18 and 22-25 in Table 1).

The analyses consisted in logistic regressions (using the ‘glm’ function of the ‘stats’ package in ‘R’; R-Development-Core-Team 2011) incorporating all fixed variables. A downward model selection based on AICc was implemented. Quadratic and cubic effects were tested for all continuous variables as well as all potential 2x2 interactions. All regressions were performed with orthogonal contrasts and type-I and III Ancova were respectively performed using the functions ‘aov’ and ‘Anova’ (package ‘car’, Fox and Weisberg 2011).

To assess how much unobserved structure of our dataset may bias our results, we performed the mixed logistic regression equivalent of the selected model, in terms of variables incorporated, with a random intercept corresponding to the individual identity or its mother’s identity (i.e., variables 26-27 in Table 1; using the ‘glmer’ function of the ‘lme4’ package ([Bates et al. 2015](#_ENREF_4)). Holding the fact that our aim was not to disentangle observed from unobserved heterogeneity, we then discussed the magnitude of the variance of the random variables as well as potential changes in magnitude and significance of the other variables’ coefficients.

**Results**

In the studied population, breeding opportunity was determined by the population manager. The choice of giving a female an opportunity to breed depended first on the size of the population at the beginning of the LD season (see Table A1.1). This ‘pseudo-density’ effect (*BOperYear*) resulted from management constraints aiming at maintaining the size of the population around 400 individuals, and not from inter-individual competition for resources or mates. The choice of giving a breeding opportunity to a female also depended on its age. Because females entered the study at their first opportunity to breed (either in LD1 or LD2) they had a probability to reproduce equal to one at these age (captured by Age^1^). Breeding opportunity then remained constant until 6 years of age (captured by Age^2^) and declined sharply after (captured by Age^3^). This was a management decision, i.e. not to allow females older than 6 to reproduce.

The choice of giving several opportunities to breed to a female depended on its past breeding failures. Population manager tended to give another chance to females failing their first attempt to breed (captured by *Failure1th*). By contrast, females succeeding their first reproduction attempt (n = 191) had a reduced chance to be offered another opportunity to breed (30%) relative to unsuccessful females (N = 80; 45%) on the next year. However, females accumulating several failures in a row exhibited a lower chance of having further breeding opportunity (captured by *ZeroSuccess*). More generally, because management tended to favour the reproduction of as many different females as possible, females that already have had the chance to breed several times had a reduced probability to breed more (captured by *cumBO*). Interestingly for further analysis, *BO* was independent of past reproductive success (*CumRS* and *CumBO*CumRS* did not improve the model) once *ZeroSuccess* was accounted for. This means that, for females that had at least one reproductive success, breeding opportunity was independent of previous reproductive success.

Population manager also considered body mass at entrance in LD seasons in the selection of breeding females (see Table A1.1). Sudden loss of mass compared to the previous reproductive season was linked to compromised health and lower chance of breeding opportunity (captured by *LossMass*). Captive females can be prone to overweigh compared with wild females (Hämäläinen et al. 2014), and females that put up some weight compared to the last time they had the opportunity to breed exhibited a reduced chance to breed (captured by *Rmass*).

Overall, the models explained 68% (McFadden pseudo R^2^, more adapted to binomial regression) to 72% (conventional R^2^) of the variance in breeding opportunity; mostly due to *BOperYear, Age, Failure1th, CumBO* and *Rmass*. Random variables on individual’s or mother’s identity had a limited but not negligible variance (respectively 0.75±0.87 and 1.4±1.18) reflecting the fact that population manager tended to favour the breeding opportunity of females from successful mother. However, this did not modify the magnitude or significance of the results (see Table A1.1).

Results concerning *RS* were more straightforward. For first reproduction, (n=271), several variables *LBOmass,* *Rmass, LossMass, CumBO, CumRS, Failure1y1th* and *Zero Success* were not incorporated into the analysis because either they had no variance (for *LBOmass,* *Rmass, LossMass, CumBO, CumRS*) or they were structurally correlated to response variables (*Failure1th* and *ZeroSuccess*). Mean reproductive success was 72% and was independent of first season at reproduction (*FirstBO*). Only *YearBirth* (p = 3.8.e^-10^) influenced reproductive success at first reproduction, explaining 25% of the variance.

Concerning further reproductive success, its variance resulted from *Failure1th* and *LossMass* (see Table A1.2). A slight senescence in reproduction was detected (effect of *Age^2^*). Hidden heterogeneity in reproductive success among individuals or sibship heterogeneity was negligible with estimated variance of random variable *Idi* and *MatEffect* being respectively 5.9e^-14^ and 2.0e^-16^. Interestingly for further survival analyses, reproductive success was independent of past reproductive successes (*cumRS*) and failures (*cumBO***cumRS*). Overall, this model explained only 24% (MacFadden pseudo-R^2^) to 28% (conventional R^2^) of the variance of reproductive success.

**References**

Bates, D., Maechler, M., Bolker, B., & Walker, S. (2015). Fitting Linear Mixed-Effects Models Using lme4. Journal of Statistical Software, 67(1), 1-48.

Fox, J., & Weisberg, S. (2011). An R Companion to Applied Regression. Thousand Oaks, CA: Sage.

Hämäläinen, A., Dammhahn, M., Aujard, F., Eberle, M., Hardy, I., Kappeler, P.M., … Kraus, C. (2014). Senescence or selective disappearance? Age trajectories of body mass in wild and captive populations of a small-bodied primate. Proceedings of the Royal Society B Biological Sciences, 281, 20140830.

R-Development-Core-Team. (2011). R: A language and environment for statistical computing. Vienna, Austria: R Foundation for Statistical Computing.

**Table A1.1** – Result of logistic regression and type-I and III- variance analysis for breeding opportunity (*BO*). Type-I and III variance analysis exhibit similar results, with variables *BOperYear*, *Age*, *Failure1th*, *CumBO* and *Rmass* explaining most of the variance in *BO* for our dataset. Variable *LossMass* has a large and significant effect but accounts for a small part of overall variance of *BO.* Together the model explains 68% (McFadden pseudo R^2^, more adapted to binomial regression) to 72% (conventional R^2^) of the variance in *BO*. Incorporating a random intercept to account for maternal effect or individual heterogeneity does not modify the magnitude or significance of the coefficients, even if quadratic and cubic effects are removed. To the opposite of what was expected, the *FreqLineage* variable did not improve the fit of the model because the frequencies of lineages are constantly increasing, constant or decreasing over the period and this constant trends are better captured by the *Lineage* variable. Variables are defined in Table 1.

|  | Logistic regression | | | | | | | Type-III Ancova | | | | Type-I Ancova | | | | + random intercept on the mother | | + random intercept on the individual | |
| --- | --- | --- | --- | --- | --- | --- | --- | --- | --- | --- | --- | --- | --- | --- | --- | --- | --- | --- | --- |
|  | Df | Estimates | SD | z -value | Pr(>\|z\|) | | | SumSq | F-value | Pr(>F) | | SumSq | F-value | Pr(>F) | | Estimates | Pr(>\|z\|) | Estimates | Pr(>\|z\|) |
| Intercept | 1 | 3.11e+01 | 3.96e+03 | 0.008 | 0.99 |  | | 2.331 | 25.11 | 6.71e-07 | *** | - | - | - | - | 2.85e+01 |  | 2.940e+01 |  |
| *BOperYear* | 1 | 6.21e+00 | 1131e+00 | 5.489 | 4.03e-08 | | *** | 3.390 | 36.51 | 2.35e-09 | *** | 23.07 | 248.463 | <2,00e-16 | *** | 5.65e+00 | *** | 6.037e+00 | *** |
| *Age* | 1 | -2.33e-02 | 5.33e-03 | -4.382 | 1.17e-05 | | *** | 4.276 | 46.06 | 2.26e-11 | *** | 44.05 | 474.506 | <2,00e-16 | *** | 6.13e-04 |  | 9.767e-04 |  |
| *Age^2^* | 1 | 2.26e-05 | 4.91e-06 | 4.535 | 5.75e-06 | | *** | 4.229 | 45.55 | 2.90e-11 | *** | 16.63 | 179.164 | <2,00e-16 | *** | ^c^ | | | |
| *Age^3^* | 1 | -6.30e-09 | 1.39e-09 | -4.535 | 5.75e-06 | | *** | 3.620 | 38.99 | 6.96e-10 | *** | 8.34 | 89.825 | <2,00e-16 | *** | ^c^ | | | |
| *Failure1th* | 1 | 2.22e+00 | 5.98e-01 | 3.709 | 2.08e-04 | | *** | 3.315 | 35.71 | 3.48e-09 | *** | 2.25 | 24.193 | 1.06e-06 | *** | 2.88e+01 | *** | 2.924e+00 | *** |
| *ZeroSuccess* | 1 | -1.65e+00 | 6.71e-01 | -2.458 | 0.014 | | * | 1.983 | 21.36 | 4.45e-06 | *** | 2.39 | 25.719 | 4.93e-07 | *** | 5.65e+00 | ** | -2.498e+00 | ** |
| *cumBO* | 1 | -2.10e+00 | 3.24e-01 | -6.478 | 9.31e-11 | | *** | 9.332 | 100.51 | <2.2e-16 | *** | 9.49 | 102.264 | <2,00e-16 | *** | 6.13e-04 | *** | -3.232e+00 | *** |
| *LossMass* | 1 | -3.71e+00 | 1.05e+00 | -3.537 | 4.04e-04 | | *** | 0.555 | 5.98 | 0.015 | * | 3.63 | 39.081 | 6.68e-10 | *** | 2.87e+01 | ** | -3.236e+00 | ** |
| *Rmass* | 1 | -1.30e+01 | 1.53e+00 | -8.533 | <2,0e-16 | | *** | 11.860 | 127.751 | <2.2e-16 | *** | 14.34 | 154.452 | <2,00e-16 | *** | 5.65e+00 | *** | -1.454e+01 | *** |
| *Lineage* | 5 | ^a^ | | | | | | 2.29 | 4.91 | 1.99e-04 | *** | 2.254 | 4.8554 | 1.25-04 | *** | ^a^ | | | |
| *YearBirth* | 13 | ^b^ | | | | | | 3.53 | 2.91 | 3.96e-04 | *** | 3.682 | 2.8326 | 3.67e-04 | *** | ^b^ | | | |

^a^ Five estimates. None of them is significant.

^b^ 13 estimates. None of them is significant

^c^ Model does not converge when a quadratic or cubic function of age is incorporated.

**Table A1.2** – Result of logistic regression and type-I and III- variance analysis for reproductive success (*RS*). Variables are defined in Table 1.

|  | Logistic regression | | | | | | Type-III Ancova | | | | Type-I Ancova | | | |
| --- | --- | --- | --- | --- | --- | --- | --- | --- | --- | --- | --- | --- | --- | --- |
|  | Df | Estimates | SD | z -value | Pr(>\|z\|) | | SumSq | F-value | Pr(>F) | | Sum Sq | F-value | Pr(>F) | |
| Intercept | 1 | -2.02e+00 | 2.18e+00 | -0.924 | 0.355 |  | 0.044 | 0.222 | 0.638 |  | - | - | - | - |
| Age | 1 | 8.61e-03 | 3.50e-03 | 2.458 | 0.014 | * | 1.206 | 6.123 | 0.015 | * | 0.075 | 0.380 | 0.53873 |  |
| Age^2^ | 1 | -3.36e-06 | 1.45e-06 | -2.333 | 0.020 | * | 1.089 | 5.528 | 0.020 | * | 0.556 | 2.823 | 0.09560 | † |
| Lineage | 5 | a | | | | * | 3.651 | 3.708 | 0.004 | ** | 2.609 | 2.650 | 0.02624 | * |
| freqLineage | 1 | 2.19e+01 | 9.79e+00 | 2.242 | 0.025 | * | 1.004 | 5.098 | 0.026 | * | 0.572 | 2.906 | 0.09093 | † |
| LossMass | 1 | -3.95e+00 | 1.34e+00 | -2.959 | 0.003 | ** | 1.940 | 9.848 | 0.002 | ** | 1.732 | 8.794 | 0.00366 | ** |
| Failure1th | 1 | -1.78e+00 | 4.72e-01 | -3.779 | 0.0001 | *** | 3.350 | 16.01 | 7e-05 | *** | 3.347 | 16.998 | 7.03e-05 | *** |

^a^ Five estimates. Two lineages ‘581LOC’ and ‘779BE' show significant decreased chances of reproductive success.
