# Supplementary material for: Female reproduction bears no survival cost in captivity for gray mouse lemurs
Source: Ecol Evol. 2019 May 18;9(11):6189–98. doi: 10.1002/ece3.5124 (PMC6580269; doi:10.1002/ece3.5124)
Supplement: Supplementary file 2 [file ECE3-9-6189-s002.docx]

**Appendix 2 – Cox model and estimation of the risk of type 2 statistical error**

**Methods**

Cox models are semi-parametric survival analyses that describe the mortality hazard *h(t|z_i_)* of individual *i* at age *t* as a function of the set of covariates *z_i_* describing the individual *i* such that:

$$h\left(t|z_{i}\right)=h_{0}\left(t\right)e^{\beta z_{i}}$$

where *h_0_(t)* is the baseline mortality hazard (when all variables are set to 0 or to reference values) and *β* is the vector of the coefficients corresponding to covariate Z.

Our model is time-varying and covariates may change at entrance into a new season. Let us denote *x(n)* the individual age at entrance into season *n*. The survival probability of individual *i* over the season *n* is given by:

$$p_{i}\left(n\right)=\exp\left[-e^{\beta Z_{i}}\int_{x(n)}^{x(n+1)} h_{0}\left(t\right)dt\right]$$

Let us now assume that an additional covariate *z** increases proportionally mortality hazard according to a coefficient *β*.* The new seasonal survival probability is:

$$p_{i}\left(n\right)p_{i}*\left(n\right)=\exp\left[-e^{\beta Z_{i}}e^{\beta*z*_{i}}\int_{x(n)}^{x(n+1)} h_{0}\left(t\right)dt\right]$$

and

$$p*_{i}\left(n\right)=\frac{p_{i}\left(n\right)p_{i}*\left(n\right)}{p_{i}\left(n\right)}=\exp\left[-e^{\beta z_{i}}\int_{x(n)}^{x(n+1)} h_{0}\left(t\right)dt\left(e^{\beta*z*_{i}}+1\right)\right]$$

The survival probability *p_i_(n)* is easily derived using the ‘predict’ function of the ‘survival’ package in ‘R’. We can then derive the additional survival probability *p*_i_(n)* according to any covariate *z*_i_* and coefficient *β*.*

Using the calculation above, we can then find the competitive survival probability *p_i_(n)* for each season-individual of our dataset described by their cumulative litter size at entrance in season *n* (*z**=*CumLitterSize_i,n_*). We can then generate datasets where individuals randomly die from this additional cause of death assuming that death occurs at the mid-interval between entrance into the season and death or censoring. Of course, seasons occurring after additional deaths are removed from the dataset.

We therefore used this procedure to generate datasets where season-individuals are randomly dying from an additional mortality risk due *CumLitterSize*. More precisely, 100 re-sampled datasets are generated for each level of additional relative risk.

**Results**

Fig A2 shows the % of p-values found respectively below 0.05 and 0.1 according to the number of additional deaths induced by additional mortality risks.

For a deleterious effect of *CumLitterSize* being detectable*,* it has first to overcome the protective trends (who ‘saved” around 10 to 20 females in our dataset). Then an additional ~25 deaths would be needed to detect a significant deleterious effect of reproduction on future survival. These thresholds correspond respectively to a mortality risk about 1.2 and 1.6 per new reproductive event (a *β*=0.6). This shows first that the observed protective trend is quite robust to an increased mortality due to reproduction. Second, in absence of this trend, an increase of mortality by 30% per reproduction would have been detected.


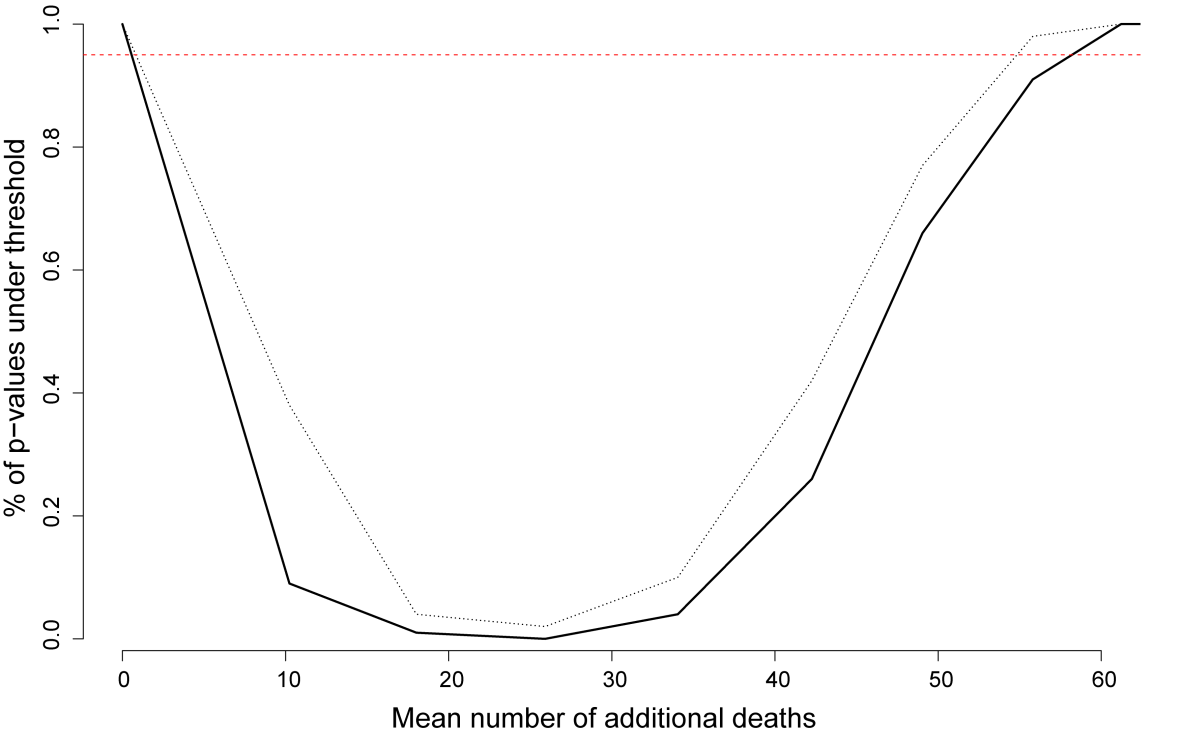


**Figure A2** – Percentage of detection of an effect of *CumLitterSize* of coefficient *β** with threshold p-values being 0.05 (plain line) and 0.1 (dotted line) according to the mean number of additional deaths randomly sampled in generated dataset. Each point corresponds to 100 randomly generated dataset for each *β** value.
