# Supplementary material for: Female reproduction bears no survival cost in captivity for gray mouse lemurs
Source: Ecol Evol. 2019 May 18;9(11):6189–98. doi: 10.1002/ece3.5124 (PMC6580269; doi:10.1002/ece3.5124)
Supplement: Supplementary file 3 [file ECE3-9-6189-s003.docx]

**Appendix 3 – Results from Preliminary Runs and selection of variables for CoR Runs**

Preliminary Runs aimed at identifying which adjustment variables together with past and current reproduction influenced females’ mortality. To do this, 65,535 models encompassing all combination of these variables were tested.

Table A3 shows the highest rank models (*ΔAICc*<2). In parallel, we also calculated covariate’s cumulative AIC weight overall the tested models (i.e. the sum of the covariates’ weight *w_i_* over all the model incorporating the covariate; with $w_{i}=e^{-0.5\Delta AIC_{i}}/\sum_{k=i}^{N} e^{-0.5\Delta AIC_{k}}$) to verify that the covariates that were overrepresented in models with the lowest AIC were also those selected within the highest ranked models. Variable cumulative AIC weights are depicted in Fig. A3.

The variables *YearBirth*, *Failure1th*, *FirstBO* and *FreqLineage* were incorporated into highest ranked models and were largely overrepresented over the models with lowest AIC. These variables were therefore unambiguously tightened to females’ mortality and potentially linked to females’ reproduction. We therefore chose to incorporate them into all models tested into CoR Runs.

The variables *RS*, *FirstBO, BOYears, CumRS* and *MassVar* had medium to large cumulative AIC weights but were not systematically incorporated into the highest rank model. We therefore chose to incorporate all combinations of these variables in CoR Runs models.

The variables *CumBO:CumRS, Mass, NoSuccess, CumBO, BO, YearObs, Lineage, MassLoss* had low cumulative AICc weights and were not incorporated into highest ranked models. We chose not to incorporate them into CoR Runs models.

Analyzing further the largest ranked models, 2x2 interactions of the variables and interaction with age at entrance into the season (‘*Age*’), or incorporating random variables accounting for frailty (p_Idi_=0.93) and maternal effect (p_Mother_=0.93) had no significant effect on mortality. Schoenfeld residuals plotted over age for each of the covariates showed no deviation from the proportional hypothesis. Martingale residuals plotted against the continuous variables *FreqLineage* and *CumRS* showed no problem in their functional distribution.

**Table A3** – The 11 highest ranked models amongst the 65,535 models tested in Preliminary Runs for which ΔAICc*<2.* Values stand for the coefficient of the variable. ‘†’ for p<0.1; ‘*’ for p<0.05; ‘**’ for p<0.01 and *** for p<0.001.

| Rank | *YearBirth* | *Failure1th* | *RS* | *FirstBO* | *FreqLineage* | *BOYear* | *CumRS* | *MassVar* | AICc | *ΔAICc* |
| --- | --- | --- | --- | --- | --- | --- | --- | --- | --- | --- |
| 1 | 0.394 ns -16.672 ns | 0.995 ** | - | -0.3 ns -1.721 ns | -1.601 ^†^ | -0.917 ^†^ | - | - | 621.23 | 0.00 |
| 2 | 0.33 ns -16.536 ns | 0.935 ** | -0.679 ns | -0.295 ns -1.696 ns | -1.66 * | -0.797 ns | - | - | 621.97 | 0.74 |
| 3 | 0.55 ns -16.534 ns | 0.882 ** | - | - | -1.451 ^†^ | -1.057 * | - | - | 621.62 | 0.39 |
| 4 | 0.435 ns -16.329 ns | 0.878 * | -0.76 ns | -0.338 ns -1.674 ns | -1.553 ^†^ | -0.871 ^†^ | - | -0.739 ns | 622.90 | 1.67 |
| 5 | 0.483 ns -16.387 ns | 0.812 * | -0.728 ns | - | -1.500 ^†^ | -0.934 ^†^ | - | - | 622.04 | 0.81 |
| 6 | 0.493 ns -16.521 ns | 0.955 ** | - | -0.34 ns -1.709 ns | -1.499 ^†^ | -0.993 * | - | -0.627 ns | 622.51 | 1.28 |
| 7 | 0.107 ns -16.537 ns | 0.827 * | -0.824 ns | -0.296 ns -1.869 ^†^ | -1.583 ^†^ | - | - | - | 622.19 | 0.96 |
| 8 | 0.579 ns -16.2 ns | 0.756 * | -0.815 ns | - | -1.417 ^†^ | -0.988 * | - | -0.73 ns | 622.94 | 1.71 |
| 9 | 0.374 ns -16.577 ns | 0.834 * | - | -0.333 ns -1.77 ^†^ | -1.755 * | -0.891 ^†^ | -0.172 ns | - | 623.12 | 1.89 |
| 10 | 0.146 ns -16.722 ns | 0.886 ** | - | -0.302 ns -1.919 ^†^ | -1.491 ^†^ | - | - | - | 622.45 | 1.22 |
| 11 | 0.639 ns -16.4 ns | 0.845 * | - | - | -1.375 ^†^ | -1.115 * | - | -0.597 ns | 622.94 | 1.71 |


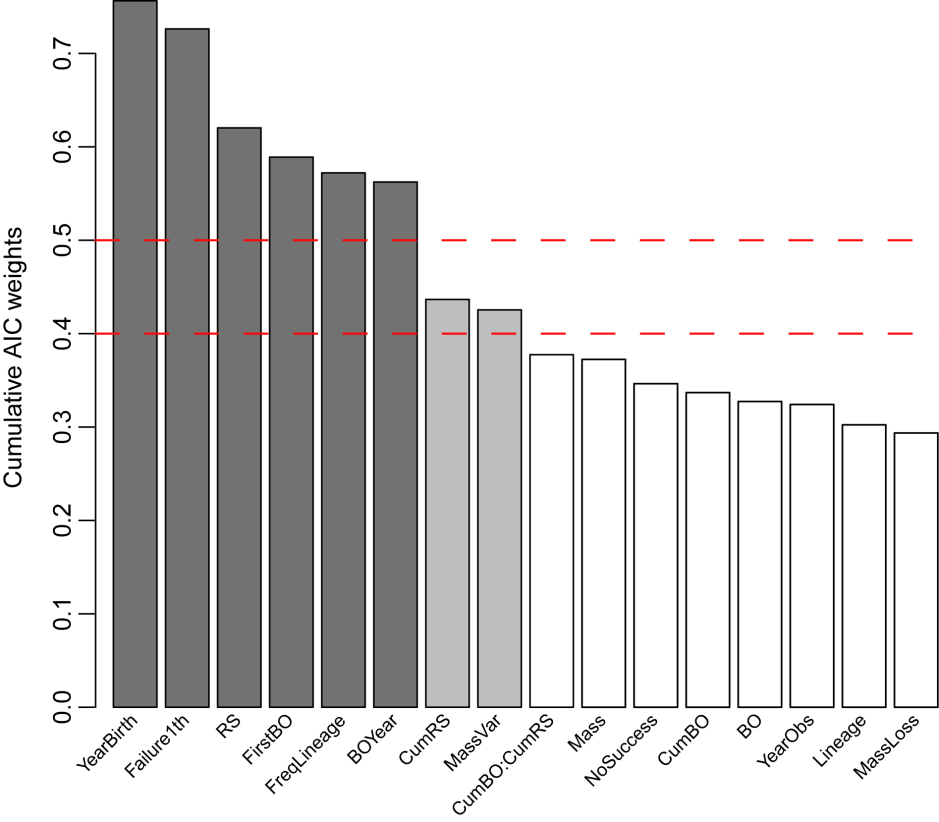


**Figure A3 –** Ordered cumulative AIC weights for variables incorporated into the 65,535 models of Preliminary Runs.
